# Serological investigation of vaccine-induced antibodies for measles, rubella, and yellow fever viruses in children vertically exposed to Zika virus or with down syndrome

**DOI:** 10.3389/fped.2023.1250059

**Published:** 2023-12-14

**Authors:** Débora Familiar-Macedo, Helver Gonçalves Dias, Fabiana Rabe Carvalho, Alex Pauvolid-Corrêa, Mayara Neto da Silveira, Mariana Cavalcante de Oliveira, Rita de Cássia Ferreira Gonçalves, Renata Artimos de Oliveira Vianna, Claudete Aparecida Araujo Cardoso, Raquel Tavares Boy da Silva, Anna Paula Baumblatt, Luzia Maria de-Oliveira-Pinto

**Affiliations:** ^1^ Laboratório das Interações Vírus-Hospedeiros (LIVH), Instituto Oswaldo Cruz, Fundação Oswaldo Cruz (IOC/Fiocruz), Rio de Janeiro, Brazil; ^2^ Laboratório Multiusuário de Apoio à Pesquisa em Nefrologia e Ciências Médicas (LAMAP), Faculdade de Medicina, Universidade Federal Fluminense, Niterói, Brazil; ^3^ Laboratório de Virologia Veterinária de Viçosa (LAVEV), Departamento de Veterinária, Universidade Federal de Viçosa (UFV), Viçosa, Brazil; ^4^ Ambulatório Multidisciplinar de Síndrome de Down (AMBDOWN), Departamento de Pediatria, Faculdade de Ciências Médicas, Universidade Estadual do Rio de Janeiro, Rio de Janeiro, Brazil; ^5^ Hospital Getúlio Vargas Filho, Fundação Municipal de Saúde, Niterói, Brazil; ^6^ Departamento Materno Infantil, Faculdade de Medicina, Universidade Federal Fluminense, Niterói, Brazil

**Keywords:** Congenital Zika Syndrome, down syndrome, immunogenicity, MMR vaccine, YF-17DD vaccine

## Abstract

**Background:**

Vaccination schedules, as well as their effectiveness and contraindications, need to be evaluated regularly, especially in specific situations. Congenital Zika Syndrome (CZS) is a severe condition that results in extensive functional and neurological impairment of fetuses and newborns due to Zika virus tropism for fetal neural progenitor cells. Down Syndrome (DS) is the leading genetic cause of intellectual disability. The immune impairment in DS has already been described, but little is known about the immune response of CZS children. Thus, CZS and DS are specific conditions that can be considered for a reassessment of the available immunizations. Here, we carried out serological analyses of attenuated vaccines-induced antibodies for measles, rubella, and yellow fever viruses in children aged 2–7, grouped into asymptomatic controls, DS children, and CZS children.

**Methods:**

Plasma samples were taken, and vaccination records were compiled during clinical follow-up. Enzymatic immunoassays for quantifying anti-measles and anti-rubella IgG were performed to assess the response to the Measles, Mumps, and Rubella (MMR) vaccine. Plaque Reduction Neutralization Test (PRNT) was performed to investigate neutralizing antibodies in response to the Brazilian vaccine strain of yellow fever (YF-17DD).

**Results:**

We highlight similar levels of anti-measles IgG and neutralizing antibodies for YF-17DD among CZS, DS, and asymptomatic children, although low positivity of measles data was seen in the three groups. In DS children, the 2–4-year-old group had an increased level of anti-measles IgG compared to the older group of children aged five to seven years. Lower anti-rubella IgG levels were observed in CZS and DS children compared to asymptomatic children. For anti-rubella IgG, the good performance of vaccination in asymptomatic children is due to younger ones rather than older ones.

**Conclusions:**

There were no reports of adverse events after the use of the MMR and YF-17DD indicating that CZS and DS could continue to receive these vaccines, but our data draws attention to the necessity of monitoring the vaccination response in CZS and DS children over time and the possible need to adhere to national measles vaccination campaigns. Scientific research needs to continue to help develop appropriate CZS and DS health guidelines.

## Introduction

Vaccine-preventable diseases such as tuberculosis, poliomyelitis, measles, and tetanus have declined considerably due to successful vaccination programs worldwide (1). Vaccination is one of the most fundamental advancements in public health as it significantly reduces mortality and morbidity rates due to various infectious diseases (2). However, five million children under 5 years old died in 2021, mostly from vaccine preventable infections (3, 4). On the other hand, there are still no vaccines for several infectious diseases and the effectiveness and contraindications of regular vaccination schedules need to be evaluated regularly, especially in specific situations, such as children affected by Congenital Zika Syndrome (CZS) and Down Syndrome (DS). Zika virus (ZIKV) can cross the maternal-fetal barrier and cause CZS, a serious condition due to the extent of functional impairment in children (5, 6). CZS is often associated with unique symptoms in infected fetuses and newborns, such as severe microcephaly with a partially collapsed skull: fine cerebral cortex with subcortical calcifications; hypertonia; macular scars and retinal changes due to viral tropism for fetal ocular and neural progenitor cells (5, 7, 8). Our group investigated a cohort of children between 2 and 3 years of age with a history of transplacental ZIKV infection. Only two of 17 children (12%) had ZIKV IgG and two of 15 (13%) had specific neutralizing antibodies for ZIKV (9, 10). This seropositivity was much lower than we expected. Based on this finding, we wondered whether these children would also have low antibody detection to other antigens unrelated to ZIKV. Salmeron et al. have shown that children with CZS react poorly to the tuberculin skin test, 2 years after BCG vaccination (11).

Another group of children that needs to be studied regarding vaccine efficacy is those with Down Syndrome (DS). Down syndrome is the most common genetic syndrome associated with intellectual disability as well as immune defects (12). The variability of the clinical presentation and the presence of clinical comorbidities associated with congenital heart diseases, digestive tract diseases, and thyroid dysfunctions directly influence the health problems experienced by these patients (13). In addition, individuals with DS often have a high frequency of infections, usually of the upper respiratory tract, characterized by greater severity and a prolonged course of diseases, which are attributed to deficiencies in the immune system (14) and to anatomical peculiarities of the face, such as shorter midface, a relative macroglossia and short eustachian tubes (12). They have substantial immune dysregulation encompassing innate and adaptive immune systems, including abnormalities in T and B cells, monocytes, neutrophil chemotaxis, circulating cytokines, and suboptimal antibody responses (15, 16). Due to these immunological disorders, they are eligible for broader vaccination coverage carried out in reference centers for immunobiological and special drugs (12, 17, 18).

The National Immunization Program (NIP) in Brazil offers 48 different immunobiological for the entire population, including children, adults, the elderly, and pregnant women (19). Among childhood vaccines, the triple viral vaccine against Measles, Mumps, and Rubella (MMR) is composed of attenuated viruses administered in two doses: the first dose at 12 months of age and the second at 15 months. At 15 months, the varicella (chickenpox) vaccine is added to the second dose of MMR (becoming MMRV). The NIP annually carries out National Vaccination Campaigns, against the antigens included in the regular Vaccination Calendar, allowing children who are not up to date with their regular vaccines to update their immunizations, and against Influenza, polio and measles (mainly observing the country's epidemiological conditions) for children under 5 years of age. Another vaccine given in childhood is the yellow fever vaccine (strain YF-17DD) which is also an attenuated vaccine given to children as young as nine months old with a booster dose at four years old (20).

Except for health complications and epidemiological risks, children with CZS and DS follow the NIP schedule (21). There is a clear need to compare the vaccination effectiveness of children with CZS and DS with asymptomatic controls so that families and health professionals can care for them with guarantees of safety in the administration of attenuated vaccines and the effectiveness of the schedule. In the present study, we sought to investigate the levels of IgG antibodies for measles and rubella vaccines and the titers of neutralizing antibodies for the yellow fever vaccine in children between 2 and 7 years old. The MMR and YFV-17DD were offered by the NIP. The data generated in this study provides us with an opportunity to assess children's ability to generate a humoral immune response to vaccines, identify possible gaps in the vaccination schedule especially in low coverage vaccination period and collaborate, based on our findings, with healthcare guidance for CZS and DS.

## Methods

### Study design

This is a cross-sectional study of convenience sampling. Between 2018 and 2022, we recruited 80 children between 2 and 7 years old. They were categorized into three groups based on the presence or absence of syndromes, which will be explained below. The asymptomatic group (*n* = 24) comprises children that do not present clinical symptoms associated with congenital syndromes due to viral infection or genetics condition. All were in good health at the time of blood collection. Some children were born to mothers who had a rash during pregnancy, and some had ZIKV detected by RT-qPCR but were born asymptomatic. All of them were negative for other congenital infections [Syphilis, Toxoplasmosis, Rubella, Cytomegalovirus (CMV) and Herpes simplex-STORCH] and to any clinical changes to date. The group of children with Congenital Zika Syndrome (CZS, *n* = 22), are those with a history of intrauterine exposure to ZIKV. These children were born from mothers who had a positive RT-qPCR for ZIKV and/or a confirmed clinical diagnosis of Zika infection during the pregnancy in the ZIKV epidemic in Brazil (2015–2016) and were negative for other congenital infections (STORCH). After birth, these children have a confirmatory clinical picture of CZS. These children are followed up at the Unidade de Doenças Exantemáticas do Hospital Universitário Antônio Pedro (HUAP) at Universidade Federal Fluminense (UFF) (Rio de Janeiro, Brazil) by Dr. Claudete Araújo Cardoso and team. The third group comprised children with Down Syndrome (DS, *n* = 34) or trisomy 21. These are children who are routinely treated at the Ambulatório Multidisciplinar de Síndrome de Down of the Hospital Universitário Pedro Ernesto (HUPE) at Universidade do Estado do Rio de Janeiro (UERJ) (Rio de Janeiro, Brazil) by Dr. Anna Paula Baumblatt and Dr. Raquel Tavares Boy da Silva. The children's vaccination records were collected after parents or legal guardians gave written consent. As an exclusion criterion, children with a history of measles, rubella, and yellow fever infection and those sick at the time or one month before blood collection were excluded from the study. According to the medical records, none of our children had history of measles, rubella, or yellow fever infection.

### Ethics statement

This study was approved by the Ethics Committee of the Oswaldo Cruz Foundation (CAAE 47358821.6.0000.5248), the Ethics Committee of UFF (CAAE 47358821.6.3001.5243 and CAAE 47358821.6.3001.524) and the Ethics Committee of UERJ (CAAE 47358821.6.3002.5259). The children's legal guardians read and signed the free and informed consent form.

### Blood samples collection

Approximately 10 ml of peripheral blood was collected via venipuncture using BD Vacutainer™ tubes containing acid-citrate-dextrose (Cat. # BD 364606). Plasma samples were obtained by centrifugation, divided into aliquots, and stored at −70 °C until analysis.

### Anti-measles and anti-rubella IgG antibody detection by commercial kit

Enzyme immunoassays (ELISA) for quantification of anti-measles (Cat. # EI 2610-9601G Euroimmun, Lübeck, Germany) and anti-rubella (Cat. # EI 2590-9601G Euroimmun, Lübeck, Germany) IgG were performed according to the manufacturer's recommended instructions. Measles IgG is expressed in International Units per liter (IU/L), where titers below 200 IU/L are considered negative, ≥200 to <275 IU/L are borderline, and ≥275 IU/L are positive. Rubella virus IgG levels are expressed in International Units per milliliter (IU/ml). Titers below 8 IU/ml are negative, ≥8 to <11 IU/ml are borderline, and ≥11 IU/ml are positive.

### YF-17DD propagation

The attenuated vaccine of yellow fever virus (YFV-17DD) strain PV027/19 was kindly provided by the Laboratório de Mosquitos Transmissores de Hematozoários (LATHEMA) of Instituto Oswaldo Cruz, Fiocruz. YF-17DD propagated in VERO cell culture (ATCC, CCL-81). Cytopathic effect (CPE) was verified daily. CPE was observed on third-day post-infection, and the culture supernatant containing the infectious virus was collected on the fifth day. Supernatant was aliquoted into working stocks, and an aliquot was subjected to RT-qPCR for YFV and other arboviruses to rule out possible contamination.

### Plaque reduction neutralization test (PRNT)

For the PRNT assay, 10^5^ VERO CCL-81 cells per well were cultured in 24-well plates and maintained in Medium 199 (Gibco, Cat.: 11150059) supplemented (10% Fetal Bovine Serum, 100 U/ml penicillin, 0.1 mg/ml streptomycin and 0.025 ug/ml amphotericin B) at 37 °C in 5% CO_2_. Plasma samples from vaccinated children were previously heated at 56 °C for 30 min to inactivate complement system proteins. Plasma samples were diluted at 2-fold serial dilution from 1:10 to 1:320. Diluted plasma samples were incubated in equal volumes with 40–80 plaque forming units (PFU) of YF-17DD strain for 1 h (at 37 °C in 5% CO_2_). After the first incubation, plasma and virus mixtures were transferred to VERO CCL-81 monolayers seeded in 24-well plates. The plates were then incubated for 1 h (at 37 °C in 5% CO_2_) with mixing every 10 min. Finally, after the second incubation, overlay solution composed by supplemented Medium 199, and 0.3% agarose was added to all wells, followed by a final incubation at 37 °C in 5% CO_2_. On the fifth day post-infection, cells were fixed in formalin for 48 h. The plates were washed, and the overlay was removed. For staining, 1% crystal violet solution was added to cell monolayers. After 1 h, the crystal violet was removed, and the plates were gently washed. Plaque forming units (PFU) were viewed and counted using a transilluminator (22, 23). Plasma samples were considered seropositive when plasma dilutions greater than 1:10 reduced 50% PFU of the YF-17DD strain. The seronegative samples reduced less than 50% PFU. In those plasma samples that had PRNT_50_ titers ≥320, no further titration was carried out.

### Statistical analysis

Descriptive and correlated analyzes were presented by medians with an interquartile range (IQR: 25%–75%) or median and minimum and maximum values, depending on the type of variable analyzed. Chi-square and Fisher's exact tests were used to assess demographics and positivity rate. Geometric means were used for PRNT titers. Kruskal–Wallis's test of variance, followed by Dunn's multiple comparison tests, was used when comparisons were made among the three groups. The Mann–Whitney test was used to compare differences between two groups or two variables within a group. Values of *p* < 0.05 will be considered statistically significant. The software used for analysis will be GraphPad PRISM version 9.01 (GraphPad Software).

## Results

### Cohort description

The demographic data of the 80 participants are presented in Table 1. The children's blood samples were taken during the clinical routine. None of the children were sick at collection or up to one month before the appointment. Asymptomatic children (*n* = 24) and children with Congenital Zika Syndrome (*n* = 22) or Down syndrome (*n* = 34) are shown. Regarding the frequency of gender or age, no difference was observed among the groups.

**Table 1:** Demographic profile of children aged two to seven years in Rio de janeiro, Brazil, 2018–2022.

Groups	Asymptomatic*^n^*^ = 24^	Congenital Zika Syndrome*^n^*^ = 22^	Down Syndrome*^n^*^ = 34^	*p*
Gender, *n* (%)				
Boys	13 (54)	15 (68)	17 (50)	0.3956
Girls	11 (46)	7 (32)	17 (50)	
Age, *n* (%)				
2–4 years	18 (75)	18 (82)	20 (59)	0.1517
5–7 years	6 (25)	4 (18)	14 (41)	

Gender and age data were represented by the number of children (*n*) and frequency (%). The chi-square test was used to assess statistical differences among groups.

#### Anti-measles IgG and anti-rubella IgG antibody levels and seropositivity in children vaccinated with MMR

**Figure 1:**
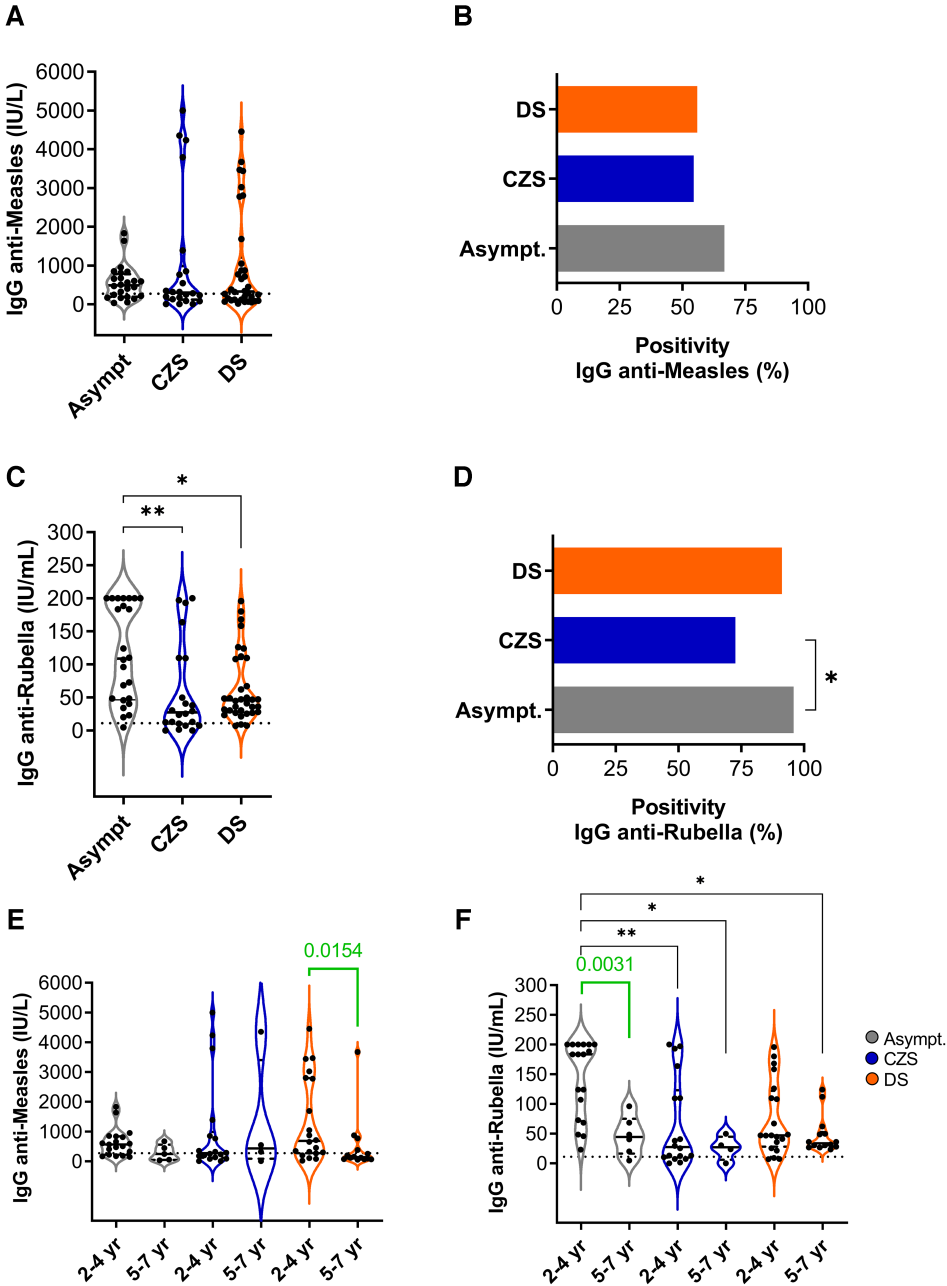
Measles and rubella IgG levels and seropositivity rate in MMR vaccinated children. (**A**) Plasma levels of anti-measles IgG (IU/L) and (**B**) frequency of measles positivity in asymptomatic, CZS and DS children. (**C**) Plasma levels of anti-rubella IgG (IU/ml) and (**D**) rubella positivity. (**E**) Anti-measles and (**F**) anti-rubella IgG plasma levels according to age group. Violin charts show the median (middle line) and describe numerical data distributions using density curves. Positive cutoffs are represented by dotted line. The Kruskal–Wallis's test was used, followed by the Dunn multiple comparison tests. Mann–Whitney test (green lines and values) assessed two groups. The asterisks indicate significant differences (*p < 0.05; **p < 0.01).

With MMR vaccination, measles and rubella IgG were quantified and expressed in IU/L or IU/ml, respectively, to assess the immune status of vaccinated children. Measles IgG was considered positive with a cutoff value of 275 IU/L or higher, as recommended by the commercial kit. Asymptomatic children had a median level of 490.1 IU/L (interquartile range [IQR]: 196.8–778.4) with 66.7% positivity (16/24), children with CZS had 302.1 IU/L (118.9–5,000) with 54.5% positivity (12/22) and children with DS had 330.2 IU/L (116.9–1,208) with 55.9% positivity (19/34). There was no significant difference in anti-measles IgG measures or positivity among groups (Figures 1A,B).

With a cutoff point equal to or greater than 11 IU/ml, the anti-rubella IgG dosage was considered positive, as recommended by the commercial kit. Asymptomatic children had a median of 108.7 IU/ml (46.5–200) with a positivity rate of 95.8% (23/24). In the CZS group, the mean IgG level was 27.5 IU/ml (9.7–109.6) with 72.7% positivity (16/22), and the DS group was 45.9 IU/ml (27.7–108.5) with 91.2% positivity (31/34). Disabled children had lower rubella IgG than asymptomatic children and lower positivity was observed in CZS compared to asymptomatic children (Figures 1C,D).

Age-based immune status is one of the factors that can interfere with vaccine efficacy. Although the measles data are similar in the three groups, children with DS aged 2–4 years old had an increased level of anti-measles IgG compared with older DS children aged five to seven years old (Figure 1E). For anti-rubella IgG, the good performance of vaccination in asymptomatic children is due to younger ones than older ones (Figure 1F). These data call attention to the need to evaluate the vaccine response of children according to age.

#### Neutralizing antibodies for YFV in children vaccinated with YF-17DD

**Figure 2:**
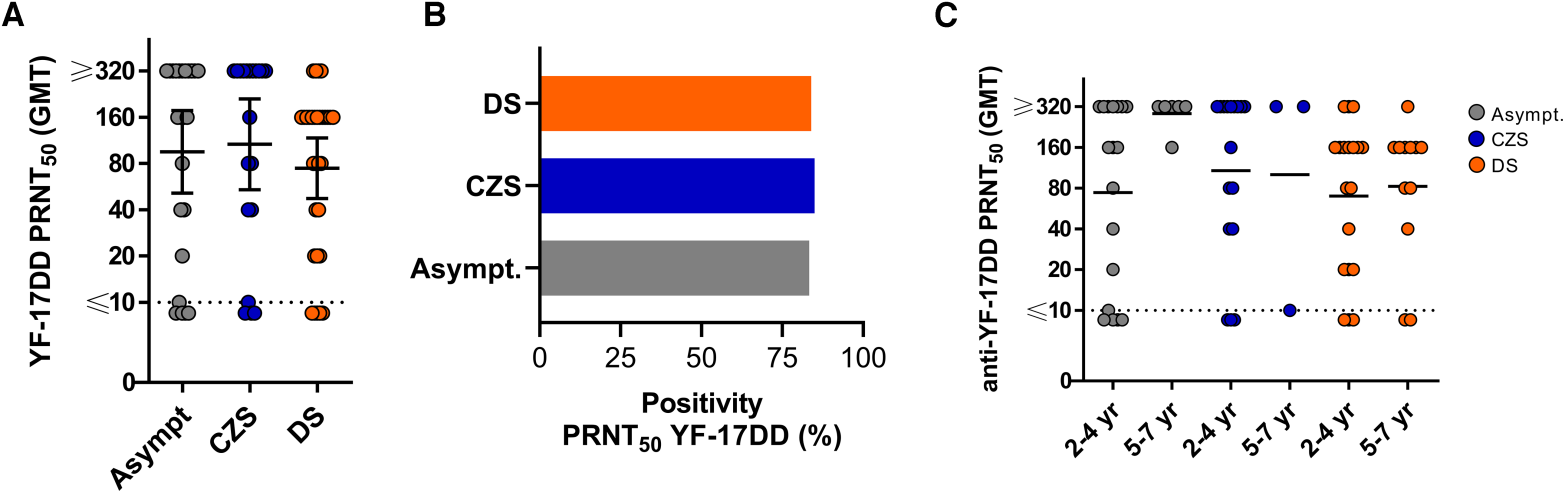
YF-17DD PRNT titers and positivity rate in YF-17DD-vaccinated children. (**A**) PRNT_50_ titers in asymptomatic, CZS and DS children. (**B**) Frequency of positivity in PRNT_50_. (**C**) PRNT_50_ titer in children by age group. Lines show the geometric mean and branches represents 95% CI. Positive cutoffs are represented by dotted line. The Kruskal–Wallis's test was used, followed by the Dunn multiple comparison tests. Mann–Whitney test assessed two groups. *p* < 0.05 were statistically significant.

To investigate the YFV-neutralizing antibody titer after YF-17DD vaccination, we used the PRNT_50_ calculation. The PRNT_50_ YF-17DD was considered seronegative when a titer cutoff value was lower than 10. This cutoff value was obtained from the assessment of three children known to be unvaccinated. The data shows that asymptomatic children had a geometric mean titer (GMT) of 95.3 (95% confidence interval [CI], 51.3–177.2), with 83.3% positivity (20/24), the children with CZS had 106.7 (53.9–210.9) with 77.3% positivity (17/22) and those with DS 74.5 (47.4–117.2) with 83.9% of positivity (26/31). It is possible that the GMT values were underestimated because our titer was up to 320. Plasma samples with PRNT_50_ titer 320 were not retested to reach endpoint titer. The data did not indicate a significant difference among groups in PRNT_50_ YF-17DD titers or positivity (Figures 2A,B). Analyzes by age groups did not show differences either (Figure 2C).

### Impact on the effectiveness of vaccination according to doses and duration of vaccination

Based on vaccination records, asymptomatic children took between two and four doses of the MMR vaccine, with a median of 27 months after the last dose, ranging from seven to 45 months. For YF-17DD, most asymptomatic children took between one and two doses as expected, but the median time elapsed after the last dose is 12.5 months ranging from six to 49 months (Table 2). These data indicate that all groups followed the national vaccination program schedule advocated by the Ministry of Health in Brazil. Regarding the number of doses or time elapsed from the last vaccination until the blood collection, we had a smaller number of children evaluated because some guardians did not take the vaccination cards with them. Thus, Table 2 indicates that the number of doses and the time elapsed between the last vaccination and blood collection were similar among the three groups. No children reported adverse events after receiving the attenuated vaccines evaluated in our study.

**Table 2:** Registration of vaccination data of children aged from 2 to 7 years old in Rio de janeiro, Brazil, 2018–2022.

Groups	Asymptomatic	Congenital Zika Syndrome	Down Syndrome	p
MMR vaccine dose	3 (2–4)*^n^*^ = 12^	2.5 (1–4)*^n^*^ = 20^	3 (1–4)*^n^*^ = 34^	0.8622
Months elapsed after the last dose of MMR	27 (7–45)*^n^*^ = 12^	32 (5–44)*^n^*^ = 20^	37 (4–61)*^n^*^ = 34^	0.0895
YF-17DD vaccine dose	1 (1–2)*^n^*^ = 12^	1 (1–2)*^n^*^ = 18^	1 (1–2)*^n^*^ = 31^	0.7335
Months elapsed after the last dose of YF-17DD	12.5 (6–49)*^n^*^ = 12^	28.5 (1–52)*^n^*^ = 18^	36 (0–64)*^n^*^ = 31^	0.0598

Vaccination data, including the number of doses and time elapsed from the last vaccination to blood collection, were described as median (min–max), and the non-parametric Kruskal–Wallis's test was used. The value (*n*) indicates the number of children evaluated.

We recategorized children according to the number of doses and duration of vaccination to assess the impact of these two factors on response to vaccine components (Table 3). Data in Table 3 was not statistically significant, however, we consider it an important description. Asymptomatic children and those with CZS who received three or more doses of MMR had a higher median measles IgG and frequency of positivity than those who received one or two doses. The three groups of children who took three or more doses of MMR showed an increase in the median rubella IgG and a higher frequency of positivity than those who took one or two doses, but without statistical difference. Children vaccinated with two doses of YF-17DD showed higher PRNT_50_ titers and positivity frequency than those who received one dose.

**Table 3:** Measles (IU/L) and rubella IgG (IU/ml) levels, YF-17DD PRNT_50_ titer, and positivity based on doses and timing of vaccination in children aged from 2 to 7 years old in Rio de janeiro, Brazil, 2018–2022.

Groups	Asymptomatic	Congenital Zika Syndrome	Down Syndrome
Measles IgG			
1–2 doses	327.5 (44.6–425.4) 60% *^n^*^ = 5^	243.1 (99.5–1,211) 40% *^n^*^ = 10^	529.1 (116.4–2,321) 62.5% *^n^*^ = 16^
≥3 doses	473.4 (189.2–675.2) 57% *^n^*^ = 7^	435 (173.3–2,101) 70% *^n^*^ = 10^	284.1 (113.8–1,208) 50% *^n^*^ = 18^
≤12 months	506.7 (297.8–744) 80% *^n^*^ = 5^	854.3 100% *^n^*^ = 1^	552.1 (250–3,196) 67% *^n^*^ = 6^
>12 months	245.5 (53.7–444.4) 44% *^n^*^ = 7^	287.6 (130–1,390) 53% *^n^*^ = 19^	307.5 (95–1,004) 54% *^n^*^ = 28^
Rubella IgG			
1–2 doses	48.5 (12.6–146.9) 80% *^n^*^ = 5^	12.9 (6.1–123.1) 60% *^n^*^ = 10^	35.8 (22.5–79) 72.2% *^n^*^ = 16^
≥3 doses	72.9 (40.6–200) 100% *^n^*^ = 7^	34.4 (21–64.7) 90% *^n^*^ = 10^	48.5 (33.3–135.4) 100% *^n^*^ = 18^
≤12 months	96.1 (70.7–191.7) 100% *^n^*^ = 5^	200 100% *^n^*^ = 1^	112.8 (45.7–171.2) 100% *^n^*^ = 6^
>12 months	40.6 (20.2–110.3) 86% *^n^*^ = 7^	25.5 (10.4–49.7) 74% *^n^*^ = 19^	36.1 (26.7–59.1) 89% *^n^*^ = 28^
YF-17DD PRNT_50_			
1 dose	160 (80–320) 86% *^n^*^ = 7^	200 (40–320) 92% *^n^*^ = 12^	80 (20–160) 82% *^n^*^ = 22^
2 doses	320 (240–320) 100% *^n^*^ = 5^	320 (280–320) 100% *^n^*^ = 6^	160 (120–240) 89% *^n^*^ = 9^
≤12 months	320 (122.1–320) 83% *^n^*^ = 6^	320 (280–320) 100% *^n^*^ = 6^	160 (160–320) 100% *^n^*^ = 6^
>12 months	240 (140–320) 100% *^n^*^ = 6^	200 (40–320) 83% *^n^*^ = 12^	80 (20–160) 80% *^n^*^ = 25^

Vaccination data, including the number of doses and time elapsed from the last vaccination to the blood draw, were described as median (25–75% interquartile) and percentage positivity (*n* of positives and rate). The non-parametric Kruskal–Wallis's test was used in three groups, and the non-parametric Mann–Whitney test in two groups. The value (*n*) indicates the number of children evaluated.

The three groups of children who received the MMR and YF-17DD vaccines up to 12 months had higher levels of IgG and PRNT_50_, in addition to a higher frequency of positivity than those who were vaccinated more than 12 months ago, but without statistical difference. Even though it was not statistically significant, this data is still encouraging.

## Discussion

Vaccination is among the best strategies to reduce infant mortality and morbidity. Even benefiting from vaccination, immunocompromised or a high-risk children's groups need to be constantly assessed regarding the immunogenicity, efficacy/effectiveness and safety of routine vaccines, especially attenuated vaccines. This article aims to compare responses to vaccines against measles, rubella, and yellow fever in children with CZS, DS and asymptomatic controls. Although there are already some studies in children with DS, there are no clear guidelines regarding vaccination in children with CZS. It is important to consider that the mortality rate in children affected by CZS is 11.3 times greater than that in asymptomatic children in the first three years of life (24). It is necessary to evaluate the safety parameters, the protective response and to monitor it, even reassessing the national vaccination calendar to discuss the need for additional doses and shorter vaccination intervals, which could translate into better vaccine responses.

The immunogenicity of some vaccines has been explored in children with DS. Kusters et al. (2011) found that DS children have a decreased anti-tetanus toxoid (TT) humoral immune response, lasting difficulties with specific anti-TT-antibody formation impaired at ages, although “protective levels” for TT are being reached (25). In the same sense, Kusters and colleagues described that only 27% of children with DS who were vaccinated against influenza A/H1N1 achieved a hemagglutination inhibition (HI) titer ≥110 after two doses of the vaccine, this titer was described as sufficient to confer conventional clinical protection (26). Another very interesting study was undertaken by Eijsvoogel et al. The authors demonstrated that children with DS present significantly lower anti-hepatitis B (anti-HBs) titers after hepatitis B vaccination when compared to control children. It is very interesting that up to 3 years of age, 100% of children had a protective anti-HBs titer, but for children over 10 years of age this titer dropped to 31.9% (27). A final study for this discussion was carried out with children with DS and their siblings. All were vaccinated against seasonal flu and received a booster dose of the pneumococcal glycoconjugate vaccine. After primary vaccination, DS children generated significantly fewer memory-specific B cells than their siblings. Following the booster dose, the response was comparable in both groups. Interestingly, specific antibody production was equally effective in SD and controls after primary and secondary immunization (28). In view of the data above published by other authors, vaccination protocols adapted to children with DS are necessary to reduce the burden of infections throughout life. To date, only Influenza, pneumococcal type 23, Varicella-Zoster (VZ), Haemophilus influenzae type b (Hib), hepatitis A (HA) and meningococcal C vaccines have been added/replaced in the routine schedule of children with down syndrome in Brazil (29). It is of great importance to evaluate in DS children the immunogenicity of other vaccines present in the immunization schedule in order to offer better guideline.

Regarding vaccination of CZS children, to date, there are no specific guideline for vaccination and there are few studies published about their immune response to vaccines. The group of Salmeron et al. reported that CZS children showed low tuberculin skin test reactivity after 2 years of BCG vaccination (11). Much like the notion of low tuberculin skin test reactivity in CZS, this response may also translate into other impaired vaccine responses.

The present study is the first to evaluate and compare the serological response in asymptomatic children to CZS and DS children after MMR and YF-17DD vaccination. A very important concern for the scientific community is the potential for immunity against all vaccine components, as is the case with MMR. As is already known and confirmed by Rasheed et al., lower levels of anti-mumps IgG were observed in young adults vaccinated with MMR vaccine compared to rubella levels (30). Because of that, here, we evaluate the response to the rubella and measles components given the epidemiological importance related to the reduction in MMR vaccination coverage in recent years that lead to the increase of confirmed cases of measles infection in Brazil and all over the world (31, 32).

After MMR vaccination, our data demonstrated an anti-measles antibody detection rate below 70% in all three groups of children. Estofolete et al. demonstrated that 60.7% and 79.8% of the children over 10 years old vaccinated with MMR had anti-measles IgG and anti-rubella IgG detected, respectively. These data were like ours, considering that 66.7% of asymptomatic control children were positive to anti-measles IgG. However, higher than those found in children with CZS, which was 54.5%, and in those with DS, which was 55.9%. Another study in Brazil demonstrated a global prevalence of 75.8% of children aged 1–15 years with antibodies against measles. Among children who had some comorbidity (immunosuppression, genetic syndrome, etc.), 72.2% had anti-measles IgG (33). The authors discuss that Brazilian children may have lower detection of IgG against measles than that observed in children from other countries, which is above 90% positivity (34, 35). The authors question the effectiveness of immunization against measles in the Brazilian population in general (36). Another previous study like ours described measles antibody seroprevalence in Mexico in 2012 and risk factors associated with susceptibility. It was a national survey representative of the general population (adults, teenagers, and children). Antibody titers against measles were evaluated by the Plaque Reduction Neutralization Test (PRNT) and classified into protective (>120 mIU/ml) or susceptible (≤120 mIU/ml) levels. The group we were interested in this study were children aged between 1 and 4 years, of which 2.18% were susceptible to measles. The authors concluded that susceptibility was associated with young age, living in Mexico City, living in crowded households, and unknown or unvaccinated status among children ages 1–5 years, and drew attention to the importance of timely administration of the first dose of the vaccine at 12 months of age (37). Therefore, seroprevalence studies in the vaccinated population are important to assess the risks, show the real epidemiological conditions and indicate the reevaluation of the current immunization program in the country.

There are few studies indicating protective limit values for IgG or PRNT antibody titers in vaccinated children for Rubella and Measles. The Rubella Subcommittee of the National Committee on Clinical Laboratory Standards in 1996 proposed the breakpoint for defining rubella immunity of 15 to 10 IU/ml. Beyond this threshold, sporadic cases of viremia and/or reinfection among previously vaccinated people with low antibody levels have been reported. Proven cases of reinfection in people with titers greater than or equal to the 15 IU/ml limit have also been reported. However, the risk is still very small compared to the high risk of rubella infection among unvaccinated people [revised by (38)]. Another study defined 200,000 International Units as the supposed level of protection for clinical measle (39). Here, the commercial kits we use indicate “antibody detection ranges”, but “the protective threshold values of IgG for measles and rubella viruses” were neither established by the kits, nor by us.

Evaluating the rubella response after the MMR vaccination, our data demonstrate that children with CZS and DS had lower IgG titers against rubella than asymptomatic control children, and lower positivity was observed in CZS (72.7%) compared to asymptomatic children (95.8%), although no statistical difference was observed in those with DS (91.2%). Our study did not demonstrate PRNT assays for measles or rubella, which may be of interest to confirm the results obtained in the ELISA assays. On the other hand, we intend to perform an additional assay on *in vitro* B cell response to measles, rubella, and yellow fever antigens to better understand the humoral response of children with CZS and DS.

Regarding the YFV-17DD vaccination, in all groups, there were children who received only one dose of YF-17DD vaccine. During the 2017 yellow fever outbreak in Brazil, the fractional dose for YF-17DD vaccine was approved for outbreak control due to its ability to induce seroconversion in seronegative participants (40). In addition, maintenance of memory CD4 and CD8 T cells and neutralizing antibodies was observed after 8 years of vaccination with a fractional dose of YF-17DD in adults (41). After ten years, individuals who received the YF-17DD vaccine still had virus-neutralizing antibodies, which motivated the World Health Organization to recommend the administration of a single dose of the YF-17DD vaccine, regardless of the individual's age (42). However, Noronha et al. showed that the proportion of seropositive and titers fell by 28% after 31–72 months and 51% after 73–100 months after the first dose, leaving a substantial proportion of children potentially unprotected (43). The authors highlight the importance of booster doses, especially in children who received the first dose in the first 2 years and reside in areas at risk for yellow fever. Following the study by Noronha et al., a second dose of YF-17DD administered at four years of age was returned to the national immunization schedule. Even so, it maintained the single dose from five years of age onwards. In the same way of MMR vaccination, the protective limit values to PRNT antibody titers in YF-vaccinated adults or children still need to be more discussed. Reis et al. conducted a retrospective study to assess the impact of age on immunity from YF-17DD vaccination. The authors considered an antibody titer ≥100 as a cutoff point and their results demonstrated that seropositivity rates were higher in adults than in children (44). Here, as we evaluated three children known to be unvaccinated by YF-17DD and they presented titers lower than 10, so we considered PRNT_50_ YF-17DD seropositive when the titer cutoff value was equal or greater than 10. We are still not confident about making a protective cut. Perhaps by using a greater number of samples, in parallel with other experimental approaches with longitudinal studies of response to vaccination, we can assume these values.

Based on the year of fraction-dose application, almost the same year that part of the children received the first YFV-17DD dose, it is possible that the families assumed that the reduced dose in children would follow the same immunological response observed in adults and, therefore, the children ended up not receiving the second dose. Furthermore, the attenuated YF-17DD vaccine is contraindicated to individuals that present congenital or secondary immunodeficiency or thymic diseases (45). As already described, 50% of DS children may have congenital heart problems that require surgical repairs (46). The thymectomy, partial or complete removal of the thymus, is usually associated with early surgical interventions for congenital heart defects in DS children (47). Here, regarding the clinical results of children with DS and consulting their medical records, 29.6% of children underwent cardiac surgery in the first years of life. We cannot guarantee that all 29.6% had their thymus compromised because we do not have access to this data. Furthermore, children with DS had median [25%–75% interquartile] values of 6,220 [5,225–8,480] leukocytes/microliter (µl), while reference values are between 3,600 and 12,000 /µl. For lymphocytes, the DS showed 2,338 [2,038–3,233] cells/µl, and the reference values are between 1,600 and 8,400 /µl. Therefore, both the median leukocyte count, and lymphocyte count in DS children were within reference values. In the case of CZS children, families reported to us during ambulatories follow-up that they found difficulties to vaccinate their children at public health centers since there is no specific protocol to follow CZS cases and YF-17DD could be contraindicated. The lack of previous experience led to limited guidance in CZS care because the knowledge continues to grow along with the children's growth (48, 49). Therefore, scientific research needs to go on to help develop appropriate healthcare guidance for CZS.

Unfortunately, the main limitations of our study were the unavailability of retrieving some medical/vaccination records information from the participants because some guardians did not have the children's vaccination cards at the time of blood collection that leads to a smaller sample size. Because of that, mainly, the understanding of the impact of doses and time elapsed since the last vaccination dose were limited. Additionally, the child recruitment was affected by the COVID-19 pandemic, leading to a smaller sample size than originally proposed. We would like to highlight that this is the first study that evaluate the serological response to MMR and YF-17DD vaccine in CZS and DS children and that this study continues, so we hope to reevaluate some points and reduce its limitations.

Our data draw attention to the need to evaluate children's vaccine response according to age. Moreover, a follow-up in rubella IgG response especially in CZS and DS children is strongly recommended. This work reinforces the need to evaluate the immune response to vaccines offered by the Brazilian national immunization program in CZS and DS children to improve the guidance in healthcare for these children.

## Data Availability

The raw data supporting the conclusions of this article will be made available by the authors, without undue reservation.
